# Comparing effects and application of telemedicine for different specialties in emergency medicine using the Emergency Talk Application (U-Sim ETA Trial)

**DOI:** 10.1038/s41598-023-40501-1

**Published:** 2023-08-16

**Authors:** Seán O’Sullivan, Henning Schneider

**Affiliations:** Faculty of Health Sciences, Technische Hochschule Mittelhessen, Gießen, Germany

**Keywords:** Medical research, Preclinical research

## Abstract

Telemedicine as a technology can support processes in the field of emergency medicine (EM) including therapies and diagnostics, but technically is often based on hardware solutions for local EM structures, especially when involving the field of pre-hospital EM. By developing an open-source, data protection compliant solution (EU GDPR and HIPAA) as well as using standardized web and open-source based technology the Emergency Talk Application (ETA) can be used as a technology that can connect emergency medical providers and include already available regional structures. By actively involving patients and connecting these with emergency or urgent care physicians ETA can be used not only as a teleconsultation system for paramedics and physicians, but in a wider network. Randomised simulation trial, comparing EM scenarios from the field of internal medicine, trauma and neurology. Participants were qualified as certified paramedics or emergency physicians (EP). Paramedics performed as ambulances crews and involved an EP if needed via ETA as Tele-Emergency Physicians (TEP). EP participated from a device of their choice, while being able to stay within their clinical workspace. From 141 scenarios 129 used ETA. Significant differences were found for the length of scenarios, duration of time the TEP was on scene, TEP arrival after scenario start, duration until TEP was called and the duration until a diagnosis was made. Also a strong positive and significant correlation between duration of the scenario and the time a TEP was bound could be described. Telemedicine is a technology that is increasingly used in the field of EM. Improving the use of telemedicine by using up-to date technology while allowing an integration of available technical and human resources is a challenge in the field of emergency medicine especially with its regional but also broad medical variety. When using one technical solution, understanding that different cases need a different medical and also telemedical approach can help in the understanding and improving therapies, diagnostics but also the involved processes and solutions. Such results are not only relevant for healthcare providers but especially by law and decision makers as to which type of solution could be introduced in each regional setting.

## Introduction

The field of Emergency Medicine is currently under pressure to develop new solutions for multiple challenges: Increasing numbers of emergencies to which ambulances respond, more patients that present themselves in emergency departments (ED) but also an aging population with increasingly complex and chronic medical histories. Furthermore, emerging diseases that can present themselves in extreme scenarios like the covid 19 pandemic are only a few but some of the reasons why the field of Emergency Medicine has to keep on evolving^1–6^.

Economic pressure, a limited number of available staff and an increasing societal urge for a quicker visit and a possible diagnosis are other reasons why new paths to provide adequate and timely treatment are more than needed^2,3,7,8^.

Also increasing accessibility to medical services by lowering boundaries especially for socially disadvantaged as well as patients from resource limited settings in an increasingly digitally connected world are challenges of our current time^9–11^.

Therefore applying novel technologies within established clinical settings could be a path to approach these challenges. Telemedicine as a digital health technology can overcome geographical distances and can connect patients with healthcare providers^12,13^.

Within the field of Emergency Medicine (EM) the use of telemedicine is as varied as the nature of the field of EM itself.

There are a variety of applications, systems and combinations that either use a store and forward technology, provide a real-time video and audio transfer (videoconference-like solutions) or even a combination of both. Also vital signs can be transmitted the same way, allowing the remote monitoring of patients (telemonitoring), while communicating with paramedics in real time and at times even the patient.

This variety allows a different delivery of telemedicine in different medical fields: The store and forward technology is often reported for the cardiovascular field, real-time video and audio transfer solutions for stroke treatment while for general and acute care a combination of both technologies is reported^14–16^.

In Addition, the availability and application of the used technology can very: For communication and visualisation, cameras can be available within ambulances, in other settings paramedics are provided with bodycams or use smartphones and tablets for communication. But also vital signs can be transferred directly, or via monitoring devices or even a data transmission unit, as well as written orders and other relevant information can be sent directly to the scene or to a T-EP. Therefore the increasing amount of options that are available in the field of telemedicine offer healthcare providers many possibilities on how treatments as well as information can be transferred, received and applied^17–23^.

Also the environment for telemedical system vary as they depends on regional emergency medical structures including geography but also on laws, data protection regulations but also on standardized medical treatment options which paramedics are allowed to provide^15–17,22,24–26^.

Therefore the concepts of telemedical system varies and so the position from which the TEP participates (that is consulted by paramedics which provide treatment) is different: The TEP can be located at an emergency dispatch center, in an other prehospital setting (like an emergency physician vehicle, like in Germany), within an ED or can be available only as a specialist for certain medical areas and questions regarding pediatrics, cardiology, palliative care, chronic or acute care but also as a general practitioner^14,27–29^.

**Figure 1:**
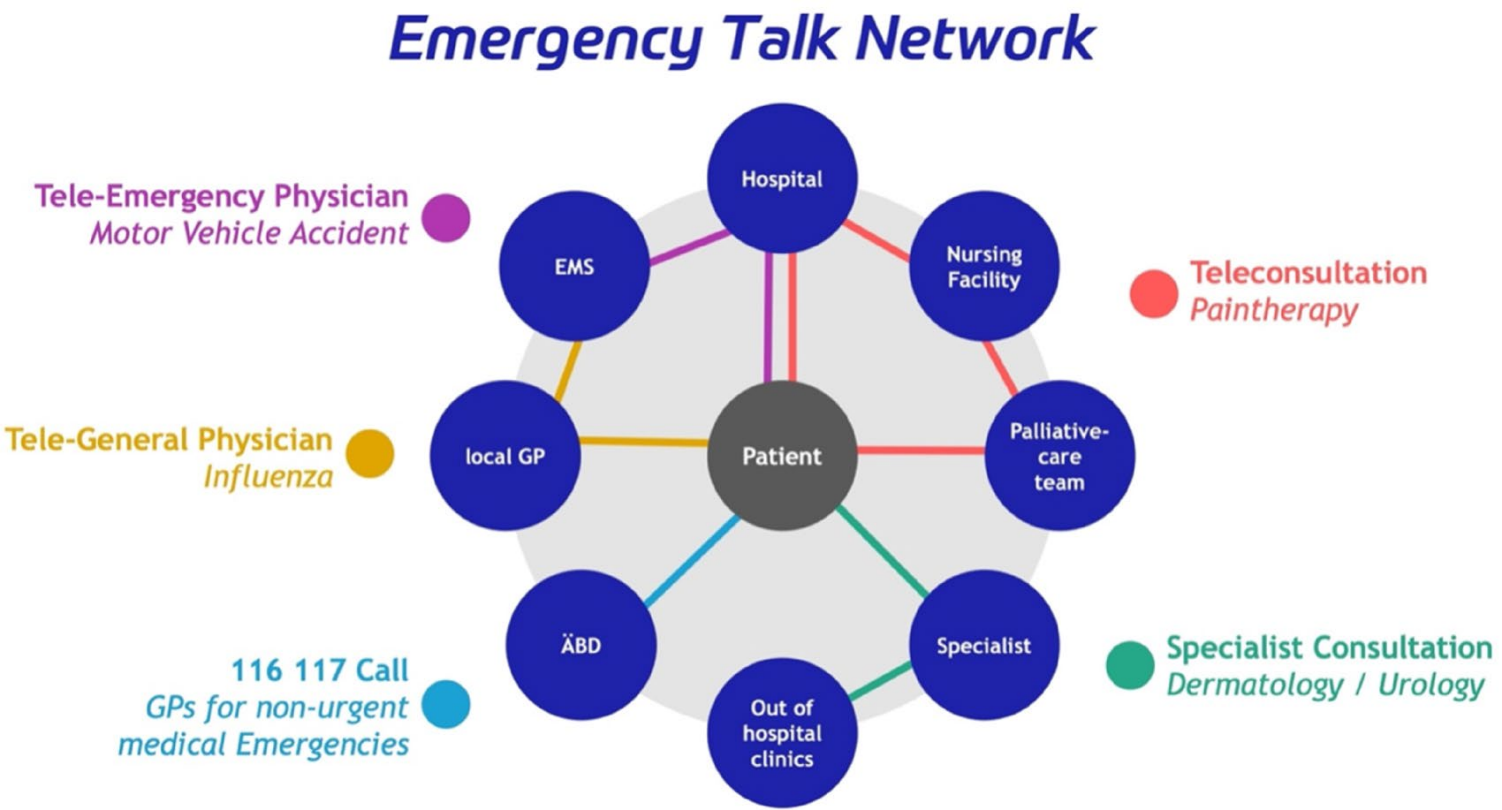
Emergency talk network: putting the patient at the center of a regional digitally supported emergency medical network.

To improve the application of telemedicine, lower the boundaries as well as improve access for low- and middle income healthcare systems we planned to provide an open-access solution so that different structures, regions and already applied concepts, could be included: By putting the patient in the center and allowing quick but safe access to a telemedical emergency network, patients can be provided with relevant medical expertise while being treated at their site of emergency. By developing an Emergency Talk Network (ETN)^30^ with a focus on the individual patient and adding technology to the already available regional structure of EM, patients might be treated quicker and more effectively. While offering non-urgent emergencies a redirection to a different healthcare provider instead of an ED and f.ex a treatment by GP or an other regionally available facility (i.e. Fig. 1).

This solution strategy builds on available regional structures and includes their strengths while adding technology to improve the patient’s pathway through the healthcare system.

This could mean that after a live telemedical consultation, patients could be referred for later treatment or if needed transferred to a hospital or specialist, if treatment via the network was not enough^28,30–32^.

Many telemedical system are used and evaluated for only one disease (like stroke or myocardial infarction) but a broader view must be taken as effectivities of emergency medical systems could be improved. Also unnecessary transports to hospitals could be reduced while improving cost-effectiveness and even reducing costs^14,15,29,33,34^.

To support this concept we developed a telemedical system called the Emergency Talk Application (ETA). This bidirection live video and audio communication solution is based on an open-source and standardized web based communication technology as defined by the W3 Consortium, while being over-compliant and encrypted with current data protection and safety standards including EU-GDPR and HIPAA^35^. This involves constantly updated security technologies that can be used with WebRTC like SSL/TLS certificates (Secure Sockets Layer/Transport Layer Security) but can also provide Transport Layer Security (TLS) like the Secure Real-time Transport Protocol (SRTP) as security threats need to be constantly monitored and managed in telemedical technologies^36^. But is also conform to different international telemedical guidelines from the field of emergency medicine like the German guideline on tele-emergency medicine or the American Telemedicine Association’s Telestroke Guidelines as well as ethical considerations in telemedicine^12,37,38^. Combining this technical and medical approach puts the patient at the center while being accessible and usable worldwide.

To test this application and concept, we focused not like other studies in the field of pre-hospital emergency telemedicine only on one tracer diagnosis like stroke, trauma or myocardial infarction, but also on common diseases like hypertensive emergencies, exacerbations of a chronic lung disease (like COPD), hypoglycemia or generalized seizures as a general application of a telemedical solution in the field of EM should be possible^32^.

## Methods

### Trial design and setting

In this first application of ETA we performed a randomized usability and simulation trial (U-Sim ETA Trial) at the Prehospital Emergency Medical Education Center Wetzlar.

As no potential harm was to be expected, the local ethics committee (University Hospital Giessen, Germany) solely required informed consent from the participants (AZ 90/21).

After written consent including a data-privacy agreement was signed, the participants received an initial lecture and introduction to ETA.

Overall there were 16 scenarios (i.e. Table 1) from the medical areas of internal medicine, trauma and neurology. These were all standardized including sequences of events, vital parameters, predefined patient answers to questions as well as complications if mistakes or a certain therapy was not performed. These scenarios are also used for the paramedic board examination in the state of Hesse. High-fidelity simulators and actors were used as standardized patients (SP), as well as standard equipment. After the introduction participants were allowed to practice with the equipment until they felt comfortable.

**Table 1:** Scenarios and their medical field.

Medical area	Scenario
Internal medicine	Anaphylactic shock Acute abdomen Acute bronchitis/COPD Acute coronary syndrome Bradycardia Diabetic emergency Hypertensive crisis Intoxication Pulmonary edema Palliative emergency Ventricular tachycardia
Trauma	Amputation of the lower limb Abdominal trauma with femur fracture Chest trauma with tension pneumothorax Burn trauma of the chest
Neurology	Status epilepticus

During the scenarios an instructor was present to enable help in case of technical problems but offered no medical support. All instructors were state certified educators for paramedic education. The teams were instructed to examine the SP like in clinical routine. If vital signs or an examination could not be performed these were announced by an instructor, but only if they were measured or examined. Observers were also present, but they were only allowed to document the scenario. Therefore comparable and uniform scenarios were ensured.

The scenario ended if the paramedics were finished with their treatment and would begin the transport of the patient. This was chosen as some participants could chose to treat the patient during a transport or within the ambulance, which could result in a lower scenario specific scoring, although an adequate therapy might have been performed “on the go”. Also a maximum time of 45 min per scenario was defined.

### Participant recruitment and randomization

Emergency medical staff and Emergency Physicians (EP) received invitations via local email lists and announcements for this simulation study and could participate voluntarily if they currently worked in the German state of Hessen.

Depending on their qualification eligibility was evaluated. Certified EMTs and Paramedics with a valid board certificate could participate. Physicians could participate if they held a valid board qualification as EPs.

After completing the registration process the participants were randomized into teams by sealed envelopes. Each Emergency Medical Service (EMS) team consisted of 2 paramedics, acting as an ambulance team and one EP, acting as the Tele Emergency Physician (TEP).

The TEP was not present at the Emergency Medical Education Center Wetzlar (trial site) but was digitally available via ETA at their clinical worksite which could be an ED, intensive care unit or a normal ward. This allowed the TEP to support the paramedics, while being able to do their regular clinical work. Participants therefore did not know the TEP before the scenario.

A week prior to the study participating TEP received a standardized introduction and practical training with the telemedicine system.

Randomization of the scenario was performed on entering the simulation room. The ambulance team pointed to one of 16 envelopes which were lying on a table. The instructor took the pointed-out envelope and took out the scenario description. The description was only visual to the instructor. Each team could then complete one of 16 randomised scenarios and not perform the same scenario more than once.

### Telemedical system

The paramedics were equipped with an Ipad Mini Series 5 (Apple Inc., California USA^39^) that had a standard browser (Safari Version 15^40^). The browsers starting page was the ETA website. Every Team received a username and a Password, so that an individual login was performed.

The TEP could use a Tablet, PC or Smartphone of their choice that was available during the time of the trial. The device had to be tested beforehand during the standardized introduction.

Once the Paramedics needed the support of a TEP, they logged into the website and chose an TEP that was shown as “available”. The TEP then received an alarm on their device. The Alarm included one of three buttons: The “accept” button allowed to accept the call right away and opened a tab in which a continuous two-way audio and video communication was directly possible. A second Tab “accept, but later” accepted the call, but opening a tab would be postponed and only a button with “available alarm” was shown. Choosing this would open a tab in which the audio and video communication was directly possible. The third button “deny” would stop and deny the alarm and no communication would be possible.

**Figure 2:**
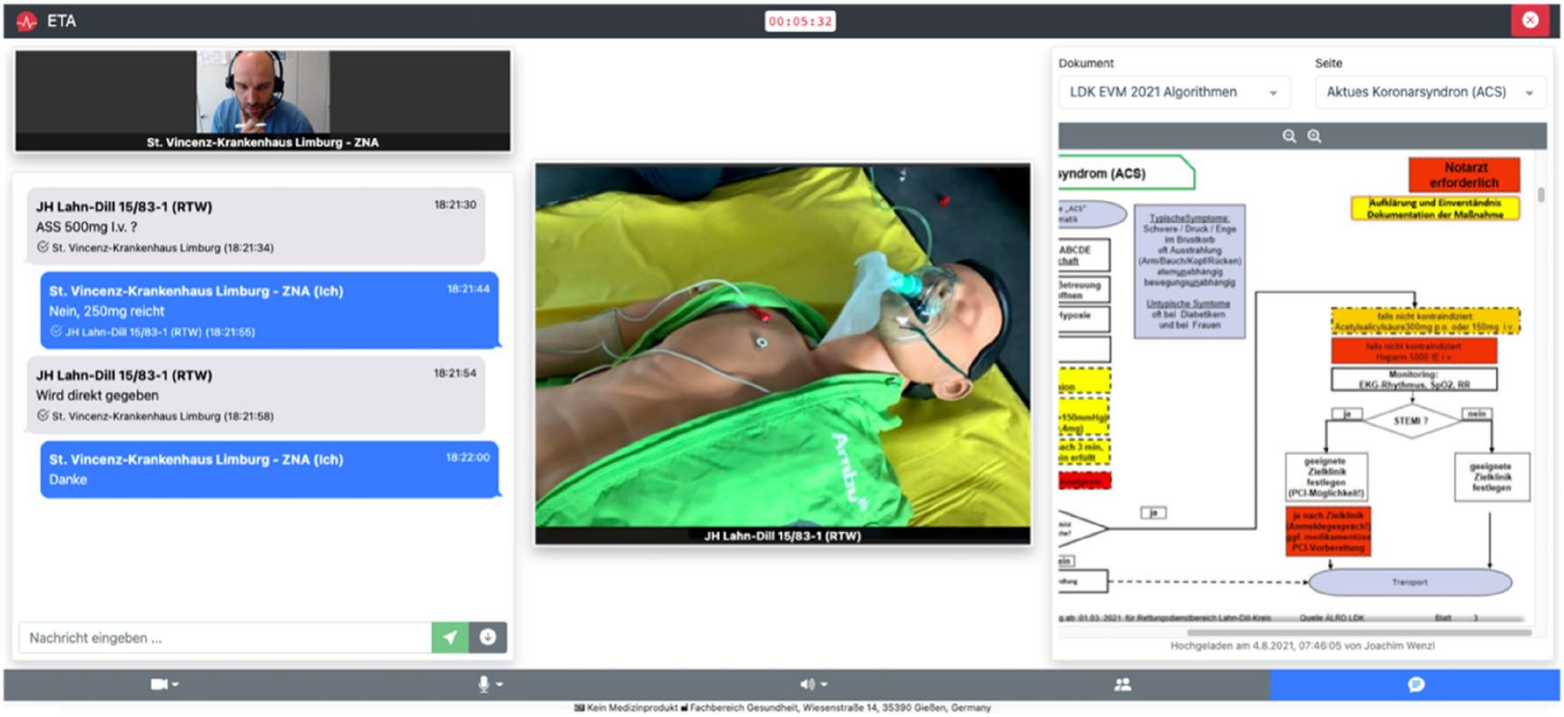
Application of ETA during a scenario.

Once the Alarm was accepted a standard screen was available for all participants (e.g. Fig. 2).

Audio and Video communication is shown in the middle. The left side allows a live messenger communication. Every sent message can be confirmed by the receiver via a “confirm” button. Every Message and confirmation has a time and date marker.

The right side shows a document viewer. The document viewer can show uploaded PDF documents like standard operating procedures or checklists. During this trial only the algorithms that were available and developed for the use within the state of Hesse were available and could be viewed by the participants^41^.

If the TEP help wasn’t needed by the paramedics anymore, he could leave by exiting the tab or window.

### Data sources and outcomes

To compare the performance of the teams a set of scenario-specific scoring items for each scenario were developed (i.e. Table 2). These items represented guideline based diagnostic and therapeutic skills which are based on medical algorithms for treatment by paramedics in the state of Hesse^41,42^. Depending on the scenario these were 8–10 skills (i.e. Supplement Table 1). These where then calculated to receive a performance percentage value (example: 7 out of 9 performed = 0.78 or 78%). This would allow a relative scenario specific comparison.

**Table 2:** Durations per medical specialty.

Duration	Internal medicine (*n* = 83)	Trauma (*n* = 33)	Neurology (*n* = 12)	p-value and effect size
Scenario length	*M* = 20:27.25 min *SD* = 4.31.93 min	*M* = 22:18.80 min *SD* = 4:17.41 min	*M* = 25:39.99 min *SD* = 8:12.34 min	*p* = 0.033 ω^2^ = 0.081
TEP on scene	*M* = 8:11.14 min *SD* = 3:30.97 min	*M* = 11:36.82 min *SD* = 5:00.12 min	*M* = 10:29.99 min *SD* = 6:35.93 min	*p* = 0.005 ω^2^ = 0.1
TEP alarm to TEP arrival	*M* = 1:51.65 min *SD* = 2:04.40 min	*M* = 2:23.18 min *SD* = 2:35.75 min	*M* = 1:26.67 min *SD* = 1:19.999 min	*p* = 0.385
Scenario start to TEP arrival	*M* = 11:18.35 min *SD* = 3:21.13 min	*M* = 9:30.45 min *SD* = 3:23.07 min	*M* = 12:55.00 min *SD* = 5:23.07 min	*p* = 0.028 ω^2^ = 0.057
Paramedic diagnosis to TEP arrival	*M* = 4:55.62 min *SD* = 3:13.82 min	*M* = 5:43.18 min *SD* = 3:51.28 min	*M* = 8:09.99 min *SD* = 6:37.86 min	*p* = 0.196
Scenario start to calling of TEP	*M* = 8:37.68 min *SD* = 4:21.02 min	*M* = 6:32.73 min *SD* = 3:44.54 min	*M* = 6:35.00 min *SD* = 5:35.98 min	*p* = 0.039 ω^2^ = 0.035
Scenario start to diagnosis	*M* = 6:07.32 min *SD* = 4:25.73 min	*M* = 3:16.36 min *SD* = 2:20.93 min	*M* = 3:45.00 min *SD* = 3:34.24 min	*p* = 0.001 ω^2^ = 0.087
Tasks and procedures	*M* = 78.3% *SD* = 13.60%	*M* = 84.19% *SD* = 13.13%	*M* = 69.05% *SD* = 5.56%	*p* = 0.001 *ω*^2^ = 0.174

Relevant time marks like the beginning, the end of a scenario, the time of diagnosis, arrival and departure of the TEP were documented.

Therefore the length of time for each scenario, time a TEP was bound, time of TEP arrival after being called by paramedics and the time until a diagnosis was made could be analyzed.

Also if a TEP was called and which participant (Paramedic or TEP) made the diagnosis was documented.

**Figure 3:**
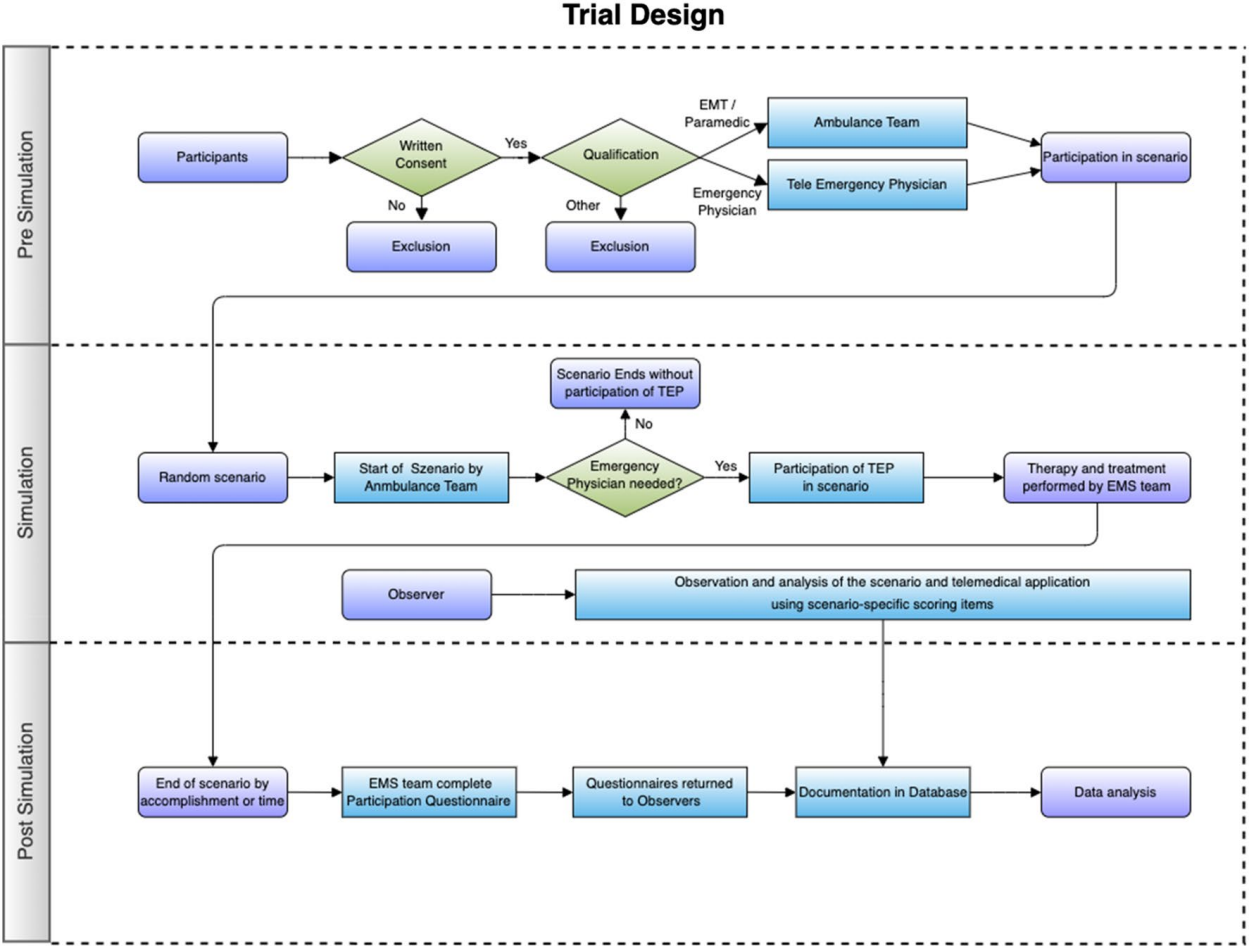
Visualization of the U-SIM ETA trial design.

Each scenario was analyzed by two faculty observers using the mentioned scoring items and chronologically documented the scenario by using a prepared form. Both observers had to reach a common decision before all data was transferred to a database (Microsoft Excel, Version 22.10, Vermont, USA^43^). Figure 3 shows the trial design.

### Statistical methods

Due to the fact that this was a pilot study and the first use of ETA, no alpha-adjustment was performed and p-values < 0.05 were considered to be significant. Categorical data were presented by frequencies and percentage.

The analysis was then performed per scenario (n = 16) and medical specialty (internal medicine, trauma or neurology; n = 3).

For the durations and scenario-specific scoring an Analysis of Variance (ANOVA) or Welch’s-ANOVA (W-test) was planned with Post-Hoc Games–Howell or Tukey analysis depending on the Homogeneity of variance (Levene’s test, *p* > 0.05.) Normal distribution was assessed by the Shapiro–Wilk test (α = 0.05) or if a sample size of n > 30 was available^44^.

If a non-parametrical test was to be performed because of a small or non-normally distributed sample the Kruskal–Wallis-Test H-Test was chosen as an alternative with Post-hoc analysis by Dunn–Bonferroni-Tests.

Omega squared (ω^2^) was used as a measure of effect size, as eta squared contains positive bias^45,46^.

Correlation analysis between scenario length and the time a TEP was bound, scenario length and scenario-specific scoring, duration of TEP bound time and scenario-specific scoring was performed by Pearson product-moment correlation coefficient (PPMCC).

If correlation analysis showed a medium to strong correlation, a regression analysis would be performed.

Regression analysis was conducted to analyse if the performance depended on the length of the scenario or the length of time the TEP was bound with a (simple) linear regression.

If more than one medium or strong correlation could be analyzed a multiple linear regression was planned. (Omega squared (ω^2^) was used as a measure of effect size).

A two-sided binominal test was performed to analyse if the diagnosis was made by the TEP or paramedics. The investigators suspected that 75% were to be made by paramedics and 25% by the TEP.

Epidemiologic data was also evaluated: Participants age, gender, experience and qualification was evaluated in an anonymized questionnaire.

The Data Analysis was then performed with IBM SPSS Statistics (Version 28.0.1.1 (14), IBM Corp., Armonk, New York, USA). Visualisation with R (Version 4.2.2 (2022-10-31), The R Foundation for Statistical Computing).

All methods were carried out in accordance with relevant guidelines and regulations. All experimental protocols were approved by the ethics committee of the Justus Liebig University Giessen (AZ 90/21).

### Ethics approval and consent to participate

As no potential harm was to be expected, the local ethics committee (University Hospital Giessen, Germany) solely required informed consent from the participants.

Participation was only possible after written consent including a data-privacy agreement was signed.

### Informed consent

Written informed consent for the publication of images and details was obtained from all participants.

## Results

### Study population

The demographic data of the 96 Participants showed some differences:

64 participated as paramedics and 32 as TEP.

Paramedics: age 22.88 (range 18–33) years, 55.26% female, 44.74% male, clinical experience 37.55 (range 12–133) months, 61.28% Paramedics, 38.72% EMTs;

TEP: age 37.06 (range 29–59) years, 33.09% female, 59.56% male, clinical experience 138.22 (range 12–360) months, 75.74% emergency physicians, 24.26% emergency physicians with a medical officers qualification (German equivalent: “Leitender Notarzt”).

Specializations were described as anesthesiology (n = 8), internal medicine (n = 8), general practitioner (n = 6), trauma surgery and orthopedics (n = 5) and neurology (n = 5).

### Oberserved outcomes

141 scenarios were performed with 129 (91.49%) having used ETA while 12 (8.51%) didn’t need the support of a TEP.

Of the 32 teams, 19 teams performed 4 scenarios (n = 76), while 13 teams performed 5 scenarios overall (n = 65).

One case in the telemedical group and one in the non-telemedical had to be excluded for not being clearly assigned to one of the 16 scenarios. Therefore 128 telemedical and 11 non-telemedical scenarios could be analyzed.

Because the amount of cases per scenario with telemedical support ranged from 7 to 12, these were grouped in to the 3 medical fields of internal medicine (n = 83; 64.32%), trauma (n = 33; 25.58%) and neurology (n = 12; 9.3%) for further analysis. With a median of 8 for internal medicine, trauma 8 and neurology 12.

Overall the amount of scenarios ranged from 7 to 10 for the field of internal medicine, trauma 8 to 10 and neurology at 13 (e.g. Supplement Table 2).

### Differences

**Figure 4:**
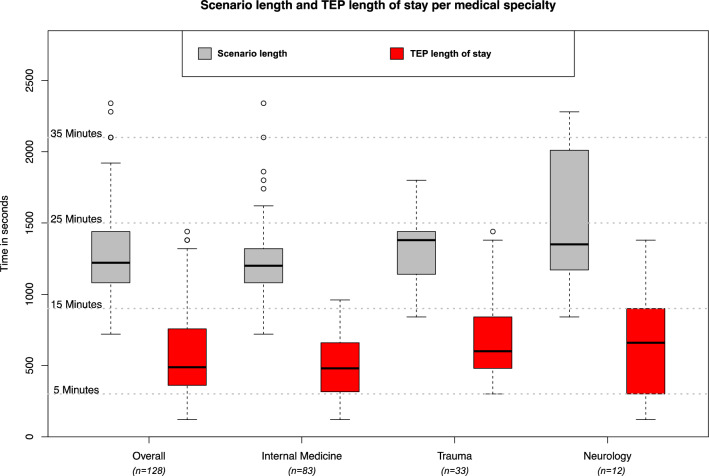
Results for scenario length and TEP length of stay overall and per medical specialty.

When comparing the medical specialties there were statistically significant differences found for the length of a scenario (*p* = 0.033), the Durations of time the TEP was on scene (*p* = 0.005), TEP arrival after scenario start (*p* = 0.028), duration until TEP was called (*p* = 0.039) and the duration until a diagnosis was made (*p* = 0.001) (e.g. Fig. 4).

When comparing task and procedures, there were also significant differences (*p* = 0.001) between the specialties (i.e. Table 2).

In the Post-Hoc analysis significant between group differences could be shown for the duration of time the TEP was on scene, from scenario start to calling of TEP, scenario start to TEP arrival and the time until a diagnosis was made. This could also be shown for Tasks and procedures (i.e. Table 3).

**Table 3:** Difference per medical specialty.

Parameter	Medical fields	Difference	95%-CI or 95% confidence interval	*p*-value
TEP on scene	Internal medicine and trauma	3:25.674 min	1:07.19 min, 5:44.16 min	*p* < 0.002
Scenario start to alarm of TEP	Internal medicine and trauma	2:04.965 min	00:08.60 min, 04:01.31	*p* = 0.039
Scenario start to TEP arrival	Internal medicine and trauma	1:47.899 min	00:07.51 min, 03:28.29 min	*p* = 0.032
Scenario start to diagnosis	Internal medicine and trauma	2:50.953 min	00:55.93 min, 04:45.98 min	*p* = 0.002
Tasks and procedures	Internal medicine and neurology	9.2503%	3.884%, 14.617%	*p* < 0.001
	Trauma and neurology	15.1443%	8.357%, 21.932%	*p* < 0.001

The previously significant result for length of a scenario (*p* = 0.033), couldn’t be shown in the following Games–Howel Post-Hoc Analysis.

### Correlation and regressions

There was a strong positive and significant correlation between duration of the scenario and the time a Tele-Emergency Physician (TEP) was bound, *r* = 0.570, *p* < 0.001 (i.e. Table 4).

**Table 4:** Correlations for durations and performed tasks and procedures.

	Scenario length	TEP on scene
TEP on scene	0.570*	
Tasks and procedures	0.008	0.166

*p < 0.001.

Between scenario length and the tasks and procedures (*r* = 0.008, *p* < 0.929) there was no correlation. Between the length of time a TEP was on scene and performed tasks and procedures was a weak but non significantly correlation (*r* = 0.166, *p* = 0.062).

**Figure 5:**
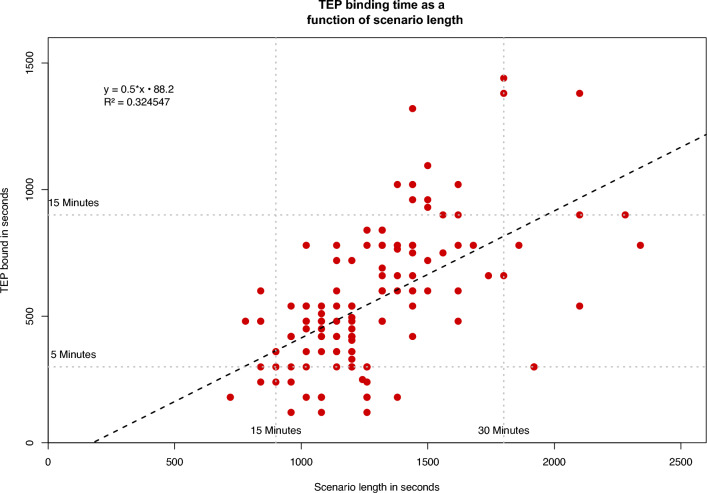
Linear regression between duration of scenario length and time the TEP was bound in seconds.

Simple linear regression was used to test if the scenario length predicted the length a TEP would be bound. The regression model was: TEP on scene = 0.502 × scenario length − 88.208. The overall regression was statistically significant (*R*^2^ = 0.32, *F*(1,126) = 60.541, *p* < 0.001) and had a strong effect (*f* = 0.68442). It was found that scenario length significantly predicted the length a TEP would be bound (β = 0*.*570, *p* < 0.001) (e.g. Fig. 5).

The exact binominal test indicated that the proportion of diagnosis made by paramedics (92.8%) was higher than the expected (75%), *p* < 0.001, *n* = 128, 2-sided.

## Discussion

The application and use of telemedicine varies in different specialties, which makes the implementation of a new solution into clinical routine and processes even more demanding in an interdisciplinary field like emergency medicine. Although there were significant differences between the different medical fields, the mean scenario length was between 20–25 min. We could show that within a few minutes paramedics could be supported by a TEP and live bidirectional communication with a patient was possible. These durations are comparable with results from other studies^18,47,48^, although this has been a simulation setting.

The support of a TEP was not needed for the whole length of a scenario but only for a short duration, while paramedics already started treatment and monitoring of the patient.

This approach allows EP to be more flexible and can therefore support more paramedics and patients in the field, by using such a sophisticated and adaptable telemedicine system like ETA. This user-based approach could therefore generally allow a more dynamic but also a specific integration of telemedicine in the field of EM allowing an improved usability of such systems.

As the landscape in pre-hospital emergency medicine differs regionally the use of telemedicine can optimize the treatment of patients and reduce the interval until a physician can perform or support the currently needed therapy to improve patient outcomes and increase clinical processes^14^.

In physician based system like Germany, in which Emergency Physician ideally arrive at the scene of emergency in an own vehicle within 15 min^49^, while in paramedic based systems, emergency physicians mainly work in Emergency Departments, the time until a patient is seen by a physician could therefore effectively be shortened to a few minutes with this technology in both systems allowing a global application of a system like ETA.

In this trial paramedics made the correct diagnosis within few minutes and initiated the needed therapy. When comparing the different medical fields like internal medicine to trauma there were more correct task and procedures performed, but this did not differ significantly between these two groups. Nor could a correlation be described in regard of the length of scenario time or longer TEP availability at the scene of emergency.

This could indicate that the quality of treatment is not automatically increased by a longer availability of a TEP or increased scenario length, but by other individual and interpersonal factors like education and experience which is a relevant issue in the use of telemedicine for clinical examination^50,51^.

When comparing the length of time until a diagnosis was made it was shown that this took longer during the internal medicine scenarios, compared to trauma, but once the TEP was on scene, this changed.

The longer stay of the TEP could have had an impact on the correct tasks and procedures during these scenarios. Usually TEP are called for further advice or to supervise more invasive procedures that paramedics aren’t allowed to perform by themselves (e. g. because of state regulations) although these are essential parts of a three-year curriculum and are lifesaving procedures^41,42^. Telemedicine could be a solution to close this gap. Supervising paramedics via telemedicine could therefore be a technology that supports these in the field, while allowing patients to safely receive lifesaving procedures as well as medication and reducing time delays in treatment^52^. Especially when rapid interventions are need like in the management of seizures improving response times and increasing diagnostic accuracy could prevent and reduce the risk of Sudden Unexplained Death in Epilepsy Patients (SUDEP)^53–55^.

In such a case, difficult airway management is clearly a field that requires specialist expertise and skills, but introduction of a supraglottic airway or bag mask ventilation could prevent hypoxia and is a skill paramedics are trained in. Regarding the application of medication by paramedics, these are only allowed to provide midazolam as a medical option for seizures in Hesse^42,56^, but an advanced medical treatment could be needed in such cases and include medication that could be provided by paramedics (e.g. levetiracetam, propofol etc.)^57^. Telemedical support and a supervision of a T-EP could increase the response times, increase the safety of correct dosages and the choice of medication, especially in special populations like children.

This shows that telemedicine is not only a solution to reduce a physician free interval, but also to supervise and safely perform therapies that manually can be performed by qualified paramedics, which national or local regulations often do not allow to be performed.

Therefore further research is needed to answer question about the safety and management of relevant clinical complications in these procedures and their management in regard of the availability of telemedical communication solutions and a possibility of supervision by T-EP^58^.

Furthermore effectively managing the limited available staff could be a reason for law-makers and healthcare policy regulators to introduce or even improve regional telemedical technologies. With solutions like ETA independent, modern, safe and GDPR conform as well as a low-cost and open-source based web technology is now available for healthcare providers and patients.

### Limitations

As this was a simulation trial, results cannot be directly transferred to clinical application. Many external, but also regional and individual factors influence the therapy of patients. But when comparing the length of time consultations were comparable with other results. Therefore an implementation of such a system in an ED must be evaluated, as this might increase the workload, availability of physicians and processes would have to be adapted^26^.

Also the qualification of paramedics varies and depends mainly on the emergency medical system, which can lead to differences in therapy options.

This was the first time ETA was used as a telemedical solution. As this solution was not compared with other systems the results cannot directly be replicated to other systems. Comparing telemedical system could allow a comparison of response times, but also in the length of stay as the medical capabilities within different systems vary.

Also for this first application of ETA we wanted to test it in the most standardized, structured and comparable field of emergency medicine, which are urgent emergencies. As the applied scenarios are standardized and approved by local healthcare authorities for the use in board examinations of paramedics in Hesse, we saw this as a way of improving standardization of medical simulations as much as possible. This involves not only the medical histories, expected and allowed therapies by paramedics, but also all the surroundings and the environment of each scenario.

But of course non-urgent emergencies are a relevant factor in EM and could be a field where telemedicine could be applied as accessible, digital and convenient solution. Such a technology could allow patient flows to be directed away from the ED or ambulance services directly to specialists, general practitioners or other healthcare providers, as overcrowding and an increasing number of visits for non-emergencies are an increasing global trend^2,3,8,15,59,60^. Therefore further testing in the field of Telemedicine has to involve non-urgent emergencies and the effects such technologies could have on regional structures and systems.

A bias towards using ETA could be seen, as many participants decided to use the telemedical system. Although telemedicine as a technology has been around for a while, a broad implementation in emergency medical systems has happened in many but not in all regions in Germany and might have lead participants to the decision to try this novel technology.

Although the sample sizes for internal medicine and trauma are not small, the design of a following study would have to allow a comparison with non-telemedically supported scenarios as well as balanced group sizes for each medical field. Especially the results for the field of neurology can only be limited to the scenario “status epilepticus”. We chose not to perform stroke scenarios as paramedics in Hesse do not require an EP for the treatment and transport of these cases. But as Quadflieg et al. describes, seizures are often mistakenly diagnosed as strokes and a telemedical consultation could result in a better diagnostic quality by supporting neurological examination, using questionnaires and video transmissions^61,62^.

Similarly paramedics in Hessen can be perform analgesia for the treatment of fractures without an EP and theoretically have the choice between six different analgetics^41,42^. Different studies have shown that analgesia can be safely performed using telemedicine but the option of having a TEP as a back-up solution can increase patient safety^63–65^.

Technical performance indicators have been described in the area of telemedicine but medically this field is also understudied and needs further research^66^.

## Conclusions

Developing a globally applicable and usable telemedical solution for emergency medicine requires high standards not only performance wise but especially on the technical side regarding data protection and usability.

Testing our developed solution, the Emergency Talk Application (ETA), in multiple different scenarios to provide information about medical performance can provide relevant information about using one solution in the interdisciplinary field of EM.

As emergency medical system are changing globally such results can provide further detailed evidence on how regional structures can benefit from telemedicine and how a possible application of the Emergency Talk Application could improve response times, quality of treatment and of patient outcomes.

### Supplementary Information

Supplementary Tables.

## Data Availability

The datasets used and/or analysed during the current study are available from the corresponding author on reasonable request.
