# Supplementary material for: Comparing effects and application of telemedicine for different specialties in emergency medicine using the Emergency Talk Application (U-Sim ETA Trial)
Source: Sci Rep. 2023 Aug 16;13:13332. doi: 10.1038/s41598-023-40501-1 (PMC10432512; doi:10.1038/s41598-023-40501-1)
Supplement: Supplementary file 1 — Supplementary Tables. [file 41598_2023_40501_MOESM1_ESM.docx]

**Supplement Tables**

Supplement Table 1: Scenario specific scoring items and score for each scenario.

Supplement Table 2: Performed amount of scenarios per medical field.

|  |  |  |  |
| --- | --- | --- | --- |
| *Nr* | *Scenario* | *Scenario-specific scoring items* | *Score* |
| 1 | Amputation of the lower limb | Clinical Examination (CE), Oxygen Delivery (O2-D), Measuring Vitalsigns (MVit), Epinehrin i.m., Epinehrin nebulised, i.v. line & Infusion, Epinehrin i.v., Cortison i.v., H1-Blocker i.v., Temperature management / preparation of transport | 9 |
| 2 | Burn trauma of the chest | CE, O2-D, MVit, i.v. line & Infusion, Non-Invasive Ventilation (NIV), 2nd i.v. & Infusion, S-Ketamine i.v., Midazolam i.v., Fluid therapy, Temperature management / preparation of transport | 10 |
| 3 | Abdominal trauma with femur fracture | CE, O2-D, MVit, i.v. line & Infusion, Pelvic Sling Application, S-Ketamine i.v., Midazolam i.v., Temperature management / Preparation of transport | 8 |
| 4 | Chest trauma with tension pneumothorax | CE, O2-D, MVit, Cardiopulmonary resuscitation (CPR) & ventilation, Needle decompression (NDe), i.v. line & Infusion, 2. NDe, Chest Seal, S-Ketamine i.v, Temperature management / Preparation of Transport | 10 |
| 5 | Anaphylactic shock | CE, O2-D, MVit, Epinephrine i.m., Epinephrine nebulised, i.v. line & Infusion, Epinephrine i.v., Cortison i.v., H1-Blocker i.v., Temperature management / Preparation of transport | 10 |
| 6 | Acute bronchitis / COPD | CE, O2-D, MVit, i.v. line & Infusion, Salbutamol nebulised, Atrovent nebulised, NIV, Cortison i.v., Morphin i.v., Temperature management / Preparation of transport | 10 |
| 7 | Akute coronay syndrome | CE, O2-D,MVit, i.v. line & Infusion, acetylsalicylic acid (ASA) i.v., Nitroglycerin (Nitro) sublingual, Morphin i.v., Heparin i.v., Temperature management / Preparation of transport, Alarm Hearthcatheterlab | 10 |
| 8 | Pulmonary edema | CE, O2-D, MVit, NIV, i.v. line & Infusion, ASA i.v., Nitro s.l., Furosemide i.v., Morphin i.v., Temperature management / Preparation of transport | 9 |
| 9 | Bradycardia | CE, O2-D, MVit, i.v. line & Infusion, Atropin i.v., 2nd Atropin i.v. , Adrenalin syringe pump, Pacemaker application, Temperature management / Preparation of transport | 9 |
| 10 | Ventricular tachycardia | CE, O2-D, MVitM, i.v. Line & Infusion, Pacemaker application, S-Ketamine / Midazolam i.v., Cardioversion, 2. Cardioversion, Temperature management / Preparation of transport | 9 |
| 11 | Palliative emergency | CE, O2-D, MVit, i.v. Line & Infusion, Morphin s.c. / i.v., Dimenhydrinate i.v., Temperature management / Preparation of transport | 7 |
| 12 | Intoxication | CE, Airway suctioning, O2-D, Ventilation, MVit, i.v. Line & Infusion, Atropin i.v., Norepinephrine i.v., Intubation, Temperature management / Preparation of transport | 9 |
| 13 | Acute abdomen | CE, MVit, i.v. Line & Infusion, Metamizol i.v., Temperature management / Preparation of transport | 5 |
| 14 | Hypertensive crisis | CE, MVit, i.v. Line & Infusion, Urapidil i.v., Temperature management / Preparation of transport | 5 |
| 15 | Diabetic emergency | CE, MVit, i.v. Line & Infusion, Glucose i.v., Fluid therapy, 2nd Glucose i.v., Temperature management / Preparation of transport | 7 |
| 16 | Status epilepticus | CE, O2-D, Midazolam nasal, MVit, i.v. Line & Infusion, Midazolam i.v., Temperature management / Preparation of transport | 7 |

Supplement Table 1 Scenario specific scoring items and score for each scenario. Abbreviations: Clinical Examination (CE), Oxygen Delivery (O2-D), Measuring Vitalsigns (MVit, Non-Invasive Ventilation (NIV), Cardiopulmonary resuscitation (CPR), Needle decompression (NDe), acetylsalicylic acid (ASA), Nitroglycerin (Nitro), intra venous(i.v.), intra muscular (i.m.)

| **Field** | **Scenario** | **Frequency** | | | **Sum** | | **AVG** | | **MED** | |
| --- | --- | --- | --- | --- | --- | --- | --- | --- | --- | --- |
|  |  | **Overall** | **+ TNA** | **- TNA** | **Overall** | **+TNA** | **per Scenario** | **+TNA** | **per Scenario** | **+TNA** |
| Trauma | Amputation of the lower limb | **8** | 7 | 1 | **36** | 33 | **9** | 8,25 | **9** | 8 |
|  | Burn trauma of the chest | **10** | 10 | 0 |  |  |  |  |  |  |
|  | Abdominal trauma with femur fracture | **9** | 7 | 2 |  |  |  |  |  |  |
|  | Chest trauma with tension pneumothorax | **9** | 9 | 0 |  |  |  |  |  |  |
| Internal medicine | Anaphylactic shock | **8** | 8 | 0 | **90** | 83 | **8,18** | 7,55 | **8** | 8 |
|  | Acute bronchitis / COPD | **10** | 8 | 2 |  |  |  |  |  |  |
|  | Akute coronay syndrome | **9** | 9 | 0 |  |  |  |  |  |  |
|  | Pulmonary edema | **8** | 8 | 0 |  |  |  |  |  |  |
|  | Bradycardia | **9** | 9 | 0 |  |  |  |  |  |  |
|  | Ventricular tachycardia | **8** | 7 | 1 |  |  |  |  |  |  |
|  | Palliative emergency | **7** | 7 | 0 |  |  |  |  |  |  |
|  | Intoxication | **7** | 6 | 1 |  |  |  |  |  |  |
|  | Acute abdomen | **7** | 4 | 3 |  |  |  |  |  |  |
|  | Hypertensive crisis | **8** | 8 | 0 |  |  |  |  |  |  |
|  | Diabetic emergency | **9** | 9 | 0 |  |  |  |  |  |  |
| Neurology | Status epilepticus | **13** | 12 | 1 | **13** | 12 | **13** | 12 | **13** | 12 |
| other | Other emergencies | **2** | 1 | 1 | **2** | 1 | **2** | 1 | **2** | 1 |
|  | Sum | 141 | 129 | 12 | 141 | 129 |  |  |  |  |
|  | Distribution | 100% | 91,49% | 8,51% | 100% | 91,49% |  |  |  |  |
|  | Evaluable *absolut* | 139 | 128 | 11 | 139 | 128 |  |  |  |  |
|  | Evaluable *in %* | 89,36% | 89,92% | 83,33% | 89,36% | 89,92% |  |  |  |  |

Supplement Table 2: Performed amount of scenarios per medical field. Abbreviations: “+ TNA” = scenario with telemedicine, “- TNA” = scenario without telemedicine, “Sum” = sum of scenarios per medical specialty, “AVG” = Average,” MED” = Median
